# Characterization of Open-Ended Coaxial Probe Sensing Depth with Respect to Aperture Size for Dielectric Property Measurement of Heterogeneous Tissues

**DOI:** 10.3390/s22030760

**Published:** 2022-01-19

**Authors:** Cemanur Aydinalp, Sulayman Joof, Ismail Dilman, Ibrahim Akduman, Tuba Yilmaz

**Affiliations:** Department of Electronics and Communication Engineering, Istanbul Technical University, Istanbul 34469, Turkey; aydinalp16@itu.edu.tr (C.A.); joof.sulayman@gmail.com (S.J.); ismaildilman@gmail.com (I.D.); akduman@itu.edu.tr (I.A.)

**Keywords:** open-ended coaxial probe, dielectric property measurement, skin tissue, sensing depth, phantom materials, broadband dielectric property retrieval

## Abstract

The open-ended coaxial probe (OECP) method is frequently used for the microwave dielectric property (DP) characterization of high permittivity and conductivity materials due to inherent advantages including minimal sample preparation requirements and broadband measurement capabilities. However, the OECP method is known to suffer from high measurement error. One well-known contributor to the high error rates is tissue heterogeneity, which can potentially be managed through the selection of a probe with a proper sensing depth (SD). The SD of the OECP is dependent on many factors including sample DPs and probe aperture diameter. Although the effects of sample DPs on SD have been investigated to some extent in the literature, the probe aperture diameters, particularly small diameters, have not been fully explored. To this end, the SDs of probes with three different apertures (0.5, 0.9 and 2.2 mm-diameters) were analyzed in this study. Probes’ SDs were first investigated with simulations using a double-layered sample configuration (skin tissue and olive oil). Next, experiments were performed using a commercial OECP with a 2.2 mm aperture diameter. The SD was categorized based on 5%, 20% and 80% DP change. Among these threshold values, a 5% DP change was selected as the benchmark for SD categorization. The findings suggest that probes with a smaller aperture size and correspondingly smaller SD should be utilized when measuring the DPs of thin and multilayered samples, such as healthy and diseased skin tissues, to increase the measurement accuracy.

## 1. Introduction

Each biological tissue has a unique set of dielectric properties (DPs) based on their molecular structure. Particularly at microwave frequencies, biological tissues can be characterized based on their water content. The interaction of the biological tissues with electromagnetic fields can be determined based on their DPs [1,2]. Therefore, tissue types or anomalies can theoretically be identified by analyzing the interaction between the field and tissue. Based on this principle, the DP characterization of both healthy and diseased tissues has been a research interest for scientists, primarily to enable the development of microwave diagnostic and therapeutic applications [3,4]. The DPs of many different tissue types, including but not limited to breast, liver and skin, were characterized with the open-ended coaxial probe (OECP) for cancer diagnosis and treatment [3,5,6,7,8].

The accurate characterization of DPs is of importance for the proper design and evaluation of microwave diagnostic and treatment modalities. The most common form of measurement technique for biological tissue DPs is the OECP due its minimum requirements for sample preparation, broadband measurement capabilities, ease of use and many other advantages. Despite the listed benefits, the OECP method suffers from high measurement inaccuracies stemming from the nature of the sample under test (SUT) or from equipment failure [9]. Tissue-related errors mainly emerge from the complex nature of biological tissue and, in some cases, the heterogeneity, surface type and size of the SUT. In others, the state of the SUT, such as ex vivo and in vivo, inconsistent tissue temperatures during the experiment and the probe–tissue interaction, such as the contact or pressure between the probe tip and tissue, can affect the accuracy. Equipment-related errors are primarily associated with measurement malpractices including the choice of a probe with an incompatible sensing depth (SD) to the tissue type, the use of a low-quality connector or RF cable between the Vector Network Analyzer (VNA) and the probe and an increase of noise levels by setting an high intermediate frequency (IF) filter bandwidth on the VNA. Part of the equipment-related errors can be minimized via system calibration. To this end, appropriate equipment selection, especially proper probe selection with a proper SD, is vital for the accurate DP characterization of the SUT. Studies on probe SD with a variety of probe aperture sizes have been reported with diverse settings under different terminologies [8,10,11,12,13,14]. In [10], two probes with different aperture sizes (2.2 and 3.58 mm-diameters) were compared for the accurate dielectric property characterization of breast tissues. The reported study in [10] was conducted using pure alcohols and de-ionized water in order to identify the sensing volume of two probes. It was concluded that the required sample thicknesses for 2.2 and 3.58 mm-diameter probes were 0.75 to 1.5 mm and 1.25 to 3.0 mm for de-ionized water and alcohol, respectively. In [11], effective penetration depths of 18 and 21 mm-diameter probes were compared. The reported effective penetration depths of 18 and 21 mm-diameter probes were 1.84 and 2.75 mm, respectively. Another comparison study for 2.2, 9.5 and 19 mm diameter probes was performed by [12], and the results showed that the sensing radius of the probes expands linearly with the inner radius of the outer conductor. It was also determined that the effect of the inner conductor on the sensing radius was higher than the insulator. In [12], the results indicated that the sensing radii for 2.2, 9.5 and 19 mm diameter probes were 1.4, 1.5, and 2.25 mm, respectively. Furthermore, in the same study, a neural network model was presented to predict the sensing radius based on the aperture size of these three probes. In [8], the dielectric property measurements of the human epidermis (palm and wrist) were performed with 3 and 1 mm diameter probes. It was concluded that the thickness of the samples should be more than 1 and 3 mm for the 1 and 3 mm diameter probes, respectively. In another study by the authors [13], the SD of the 2.2 mm diameter probe was investigated using two double-layered configurations mimicking the skin tissue heterogeneity. The SD was determined based on three thresholds of percentage increases of the measured DPs. The obtained results suggested that the DP contrast between the two layers affects the SD. That is, high contrast causes a rapid change, while low contrast causes a slower change in the measured DPs. In yet another study by the authors [14], the SD of a 2.2 mm diameter probe with a double-layered configuration using ex vivo rat breast and skin tissues were characterized. It was concluded that any membrane layer with a 0.4 to 0.8 mm thickness on the SUT can potentially impact the dielectric property measurement results by 52% to 84%.

Despite the many studies that have attempted to characterize the SD of the OECP with respect to different definitions—that is, radius, tissue and frequency—there is still a need to define the SD of probes with small aperture radii. The use of an appropriate probe dimension is of importance for the accurate DP measurement of thin multilayered-tissues such as skin tissue. This approach can potentially eliminate equipment and tissue -related errors. Therefore, the aim of this work is to investigate the SDs of OECPs with small aperture radii in order to achieve the following:

- To define the SDs of OECPs with small radii to enable the selection of appropriate probes during the DP characterization of highly heterogeneous, skin-like tissues composed of multiple thin layers;
- To form an SD definition via the examination of the three different percentage change thresholds in retrieved/measured DPs for different probe dimensions;
- To investigate the significance of tissue layers located immediately at the probe aperture during SD characterization—e.g., how thin and high DP contrast layers positioned on top of the tissue affect the probe SD.

To this end, an experiment was designed to analyze the sensing depth of the 2.2 mm diameter probe with a double-layered sample. The double-layered configuration consists of skin tissue and olive oil as the first and second layers, respectively. Three probes with different aperture sizes (2.2, 0.9 and 0.5 mm outer diameters) were simulated using Ansys HFSS 2020 R2 software (Customer Number: 316232, Canonsburg, PA, USA) using a similar double-layered configuration to mimic a heterogeneous multilayered sample. Next, the SD of each probe was characterized based on both simulation and experimental results.

The remainder of this paper is organized as follows: in Section 2, the simulations and experiments are described in detail. Section 3 shows the experimental results obtained by the 2.2 mm diameter probe based on 5%, 20% and 80% increases in the measured DP of olive oil from a 0.5 to 8 GHz frequency range. A similar method is applied to simulate the response of 2.2, 0.9 and 0.5 mm diameter probes. In Section 4, the sensing depth analysis is presented based on the probe’s aperture, and finally the conclusions are drawn in Section 5.

## 2. Materials and Methods

In this section, the simulations and experimental setup are explained in detail. Simulations with three probes along with DP retrieval from the S-parameter response via in-house algorithm are given in Section 2.1. The double-layered sample configuration and the measurement protocol are described in Section 2.2.

### 2.1. Simulation Configuration

**Figure 1:**
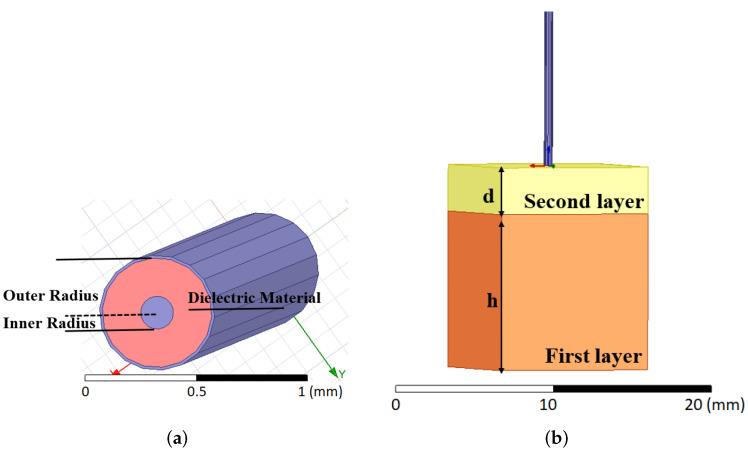
Simulated open-ended coaxial probe and configuration of double-layered sample. (**a**) Open-ended coaxial probe. (**b**) Double-layered sample, with the first layer being skin tissue with d${}_{1}$ thickness and the second layer being olive oil with d${}_{2}$ thickness.

The open-ended coaxial probes with three different aperture sizes terminated with double-layered configurations were simulated with Ansys HFSS 3D electromagnetic (EM) software (Canonsburg, PA, USA). As shown in Figure 1a, the open-ended coaxial probes were composed of two conductors with a dielectric material sandwiched in between. A commonly used dielectric material PTFE ($\epsilon_{r}$ = 2.1) was implemented in the simulations of the probe. Since the desired outer radii of probes were known, the inner radius were calculated to fix the probe input impedance to 50 Ω. The length of probes was assigned as 40 mm in order to optimize the computing process. The final dimensions of the probes are listed in Table 1. S-parameters of all probes were simulated with double-layered configuration composed of skin and olive oil. The dielectric properties of the materials used for the double-layered configuration at 4 GHz are given in Table 2. The effects of the probes’ aperture size were analyzed by changing the second layer thickness (d) as illustrated in Figure 1b.

The simulations were performed from 0.5 to 20 GHz with 0.25 GHz increments. To adjust the distance between the probe tip and first layer, the thickness of the second layer (d), shown in Figure 1b, was varied. The second layer thickness (d) was selected in three different ranges: 0.01–1 mm with 0.05 mm increments, 1–3 mm with 0.2 mm increments and 3–5 mm with 1 mm increments. These step sizes and ranges were selected to reduce simulation time and to provide the thicknesses in which essential changes in dielectric properties occur. These ranges and increments were not linearly or logarithmically determined. It should be noted that, while the thickness of the second layer determined the distance between the probe aperture and the first layer in the simulations, the probe was immersed to the second layer to adjust the distance between the aperture and the first layer during measurements. Finally, to retrieve complex dielectric properties from S-parameters obtained from simulation, Debye relaxation model parameters were iteratively computed with the in-house algorithm described in [13].

### 2.2. Experiment Setup

**Figure 2:**
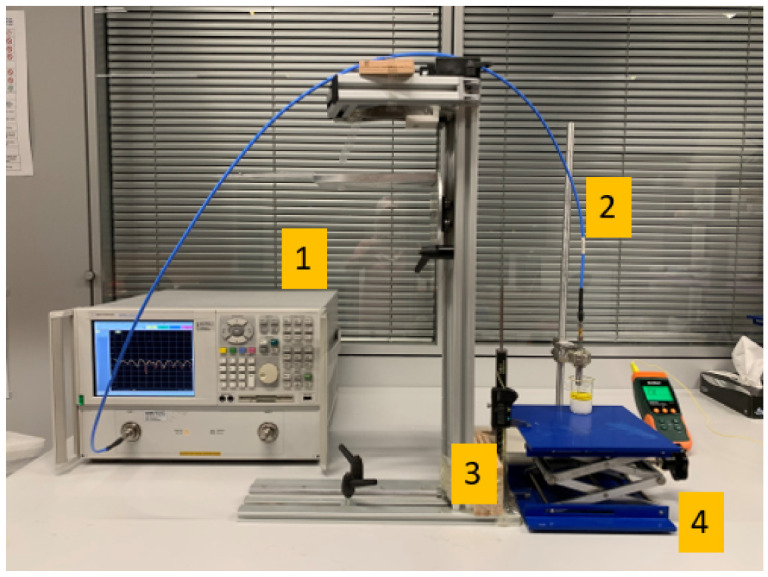
Experimental setup: (1) Agilent N5230A PNA-L Series Network Analyzer, (2) Agilent Slim Form Probe (2.2 mm-diameter aperture size), (3) digital caliper and (4) an adjustable stand.

All DP measurements reported in this work were performed with the Agilent N5230A PNA-L Series Network Analyzer (Santa Clara, CA, USA) and commercial Agilent slim form probe kit (Santa Clara, CA, USA) using the 85070E DP measurement software. The experiment setup is shown in Figure 2. Before the measurements were performed, the system was calibrated based on the software’s three-step standard calibration procedure; that is, open circuit, short circuit and de-ionized water measurement, respectively.

During the measurements, an adjustable stand and a digital caliper (Mitutoyo absolute digimatic caliper 0–150 mm with a 0.01 mm digital step size) were used to adjust and record the position of the double-layered samples. In the experimental setup, the positions of the caliper and probe were firmly fixed in order to minimize the error caused by any movement. It should be noted that the VNA was turned on 4 h before the measurements in order to minimize error due to drift in VNA measurements.

### 2.3. Measurement Samples and Protocol

The sensing depth analysis was performed with the double-layered sample consisting of a skin-mimicking phantom and olive oil. The measured dielectric properties of the pure layers are listed in Table 3. Characterization of the skin-mimicking phantom, used as the bottom (first) layer in the measurement sample along with phantom recipe, is described in [17]. Once the phantom was formed and placed in the measurement beaker, it was kept at room temperature (24 ± 2 ${}^{\circ }$C) for five days until it was solidified. Next, the double-layered configuration, shown in Figure 2, was formed by adding the olive oil as the upper (second) layer. Olive oil is a liquid with low dielectric properties that can provide a realistic contrast in dielectric properties when used as a mimicking layer to represent the effect of the keratin layer on the skin. Due to its high viscosity and non-solvent properties, it was possible for us to form two separate layers using the olive oil and phantom while preventing the mixing on the boundary of two materials. Additionally, olive oil is transparent, which allowed us to clearly inspect the movement of the probe inside the layer by eye. During dielectric property measurements, the steps given in [13] were followed.

## 3. Results

In this section, the SDs of all three probes with 0.5, 0.9 and 2.2 mm diameter aperture sizes were determined. The axial electric field magnitudes obtained from simulations using three probes with different apertures and a double-layered configuration are given in this section. Furthermore, the analyses were conducted with the relative permittivity obtained from simulation and experimental results by selecting various frequency points. The percentage changes in the relative permittivity of the double-layered configuration were examined at each frequency point with respect to the second layer thickness below the probe tip (d).

### 3.1. Electric Field Distribution

**Figure 3:**
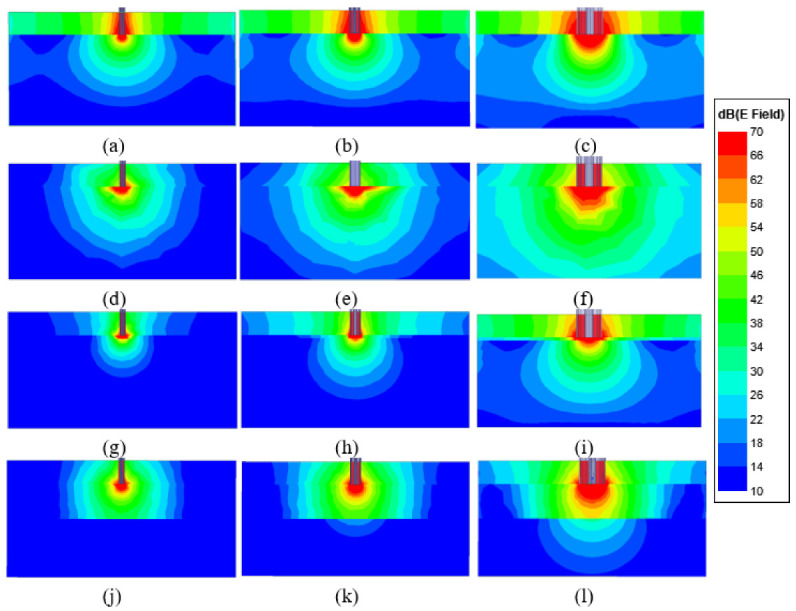
The electric field strength with 0.5, 0.9 and 2.2 mm diameter probes placed against single layer: (**a**–**c**) skin tissue layer, (**d**–**f**) olive oil layer, and double layer configurations: (**g**–**i**) olive oil layer with thickness of 0.3 mm and (**j**–**l**) olive oil layer with thickness of 3 mm.

To understand the field behavior around different probe aperture tips with different sample types, simulated axial electric field magnitudes at 2 GHz are shown in Figure 3. Figure 3a,d,g,j represent the analysis of the 0.5 mm diameter probe with a bare skin tissue layer, with only an olive oil layer, a double-layered configuration with a 0.3 mm olive oil layer and a double-layered configuration with a 3 mm olive oil layer, respectively. Similar analyses are presented in Figure 3b,e,h,k for the 0.9 mm diameter probe and Figure 3c,f,i,l for the 2.2 mm diameter probe. The simulation results indicate that the electric field distribution on the single-layer configurations differed from the double-layered configurations.

The field distribution was approximately 70 dB around the three different probe tips, and the magnitude of the field gradually decreased to 10 dB at distant points from the probe tip. As seen in Figure 3g, when the thickness of the olive oil layer was equal to 0.3 mm, the electric field distribution in both layers (olive oil and skin tissue) was found to be around 70 dB for the 0.5 mm diameter probe. However, when the thickness of the olive oil layer was increased to 3 mm, the electric field distribution on the skin layer decreased to 10 dB as shown in Figure 3j. Using the same double-layered sample, the thickness of the olive oil layer was set to 3 mm, and the simulation results for the the 0.9 mm diameter probe indicate that the field distribution on the skin layer fluctuates between 18–10 dB as seen in Figure 3k. Lastly, the simulation results for the 2.2 mm diameter probe when the thickness of olive oil layer was set to 3 mm show that the field distribution on the skin layer changes between 30–10 dB, as depicted in Figure 3l. The maximum electric field distributions at the skin tissue layer for each probe with a double-layered configuration were 10, 18 and 30 dB for the 0.5, 0.9 and the 2.2 mm diameter probes, respectively. Having a higher value of the electric field distribution at a deeper position is one of the factors affecting SD. Based on these results, the electric field distribution of the 2.2 mm diameter probe at a distant point from the aperture is higher when compared to the electric field strength of the 0.9 and 0.5 mm diameter probes, indicating that the SD of the 2.2 mm probe is larger.

### 3.2. Simulation with 2.2 mm Diameter Probe and Experimental Results

Analyses of the 2.2 mm diameter probe through simulation results were performed by evaluating the retrieved relative permittivity based on three threshold values of 5%, 20% and 80% with respect to the change in thickness of the second layer (distances from the first layer (d)). The percentage changes and corresponding distances from the first layer (d) at five frequency points 0.5, 2, 4, 10 and 20 GHz are given in Table 4.

Obtained results for the five frequency points indicate that the retrieved relative permittivity of olive oil increased by 5%, 20% and 80% between 0.66 mm to 1 mm, 0.36 to 0.86 mm and 0.16 to 0.36 mm thicknesses, respectively.

To investigate the SD of the probe experimentally, five frequency points were chosen between 0.5 and 8 GHz. Similar to the simulations, three percentage increases (5%, 20% and 80%) in the measured relative permittivity with respect to the dielectric properties of pure olive oil ($\epsilon_{r}$ = 2.67 at 4 GHz) were recorded. As shown in Table 5, a 5% increase in the relative permittivity of olive oil was observed at 0.81 mm for the five frequency points. By moving the adjustable stand upwards, the thickness of the second layer (olive oil) was decreased and the measured relative permittivity increased by 20% at 0.67 mm. Additionally, an 80% increase in measured relative permittivity was obtained when the thickness of the second layer was between 0.20 and 0.22 mm.

The simulation results given in Table 4 indicate that the frequency has an impact on the SD, represented by the percentage increase in relative permittivity according to the thickness of the second layer. However, a similar effect on SD was fizbd with respect to thicknesses in the experimental results due to the limitation of the frequency range (0.5–8 GHz). In order to prevent the cable loss, and due to equipment limitations, the frequency was limited to the indicated band. The power level of signals was set to 0 dBm. Regarding the noise reduction during measurements, with the IF bandwidth set to 10 Hz, the noise level was significantly reduced and the sweep time was 18 s. Each measurement was acquired after five sweeps, which is 18 × 5 s. It should be noted that under experimental conditions, it is expected that the measurement sensitivity will be diminished and it will be heightened during DP calculation. Thus, it can be stated that the disagreement could be due to noise during measurements. In [11,18], the sensing depth was investigated based on the frequency. In [11], based on the simulation results, it was concluded that frequency dependence occurs above 6 GHz, and [18] stated that frequency dependence can be discussed based on the materials’ characteristics by analyzing experimental results. In addition, the results obtained from our simulation show the critical changes observed from 0.5 to 4 GHz are 0.82, 0.62 and 0.27 mm for 5%, 20% and 80% increases in relative permittivity, respectively. Similarly, we acquired approximately the same thicknesses in the experiment results for 5%, 20% and 80% increases at the same frequency points.

### 3.3. Simulation Results for 0.9 mm-Diameter Probe

A second set of simulations was performed with the 0.9 mm diameter probe. Sensing depth analyses were carried out for 0.5, 2, 4, 10 and 20 GHz frequency points. In Table 6, the thicknesses (d) are listed when the change in the retrieved relative permittivity of second layer reached 5%, 20% and 80% increases at the five frequency points. It was observed that the retrieved relative permittivity with the 0.9 mm diameter probe increased 5%, 20% and 80% between 0.51 to 0.66 mm, 0.21 to 0.26 mm and 0.06 to 0.11 mm distances, respectively, at five frequency points.

### 3.4. Simulation Results for 0.5 mm-Diameter Probe

The final set of simulations was performed with the 0.5 mm diameter probe. In Table 7, the thicknesses where the change in the retrieved relative permittivity of the second layer reached 5%, 20% and 80% increases with the 0.5 mm diameter probe are listed at five frequency points. As seen from Table 7, the retrieved relative permittivity of olive oil increased by 5%, 20% and 80% at 0.31, 0.11 and 0.06 mm distances, respectively.

## 4. Discussion

SD analysis based on the OECP’s aperture size is important for choosing an appropriate probe to minimize tissue and equipment-related errors. In this context, several studies have been presented in the literature. A comparison of previously reported work on OECP SD is given in Table 8. The results (depth (mm)) given in the table are based on the definition or terminology used. Furthermore, the type of data acquisition method (experiment (Exp.) and simulation (Sim.)), the size of the probe aperture (Aper. Size (mm)), the type of sample configuration (Sam. Config.) and the relative permittivity of the samples (Sample’s $\epsilon^{\prime }$) are also given in Table 8. In the literature, aperture sizes larger than 1 mm were investigated. In this study, the sensing depths of three different probe apertures are compared to assist in the equipment and tissue-oriented design of the experimental setup. To this end, 0.5, 0.9 and 2.2 mm diameter probes and a double-layered material to represent tissue heterogeneity were designed using HFSS simulation software. Furthermore, the commercially available 2.2 mm diameter probe was utilized to validate the simulation results.

**Figure 4:**
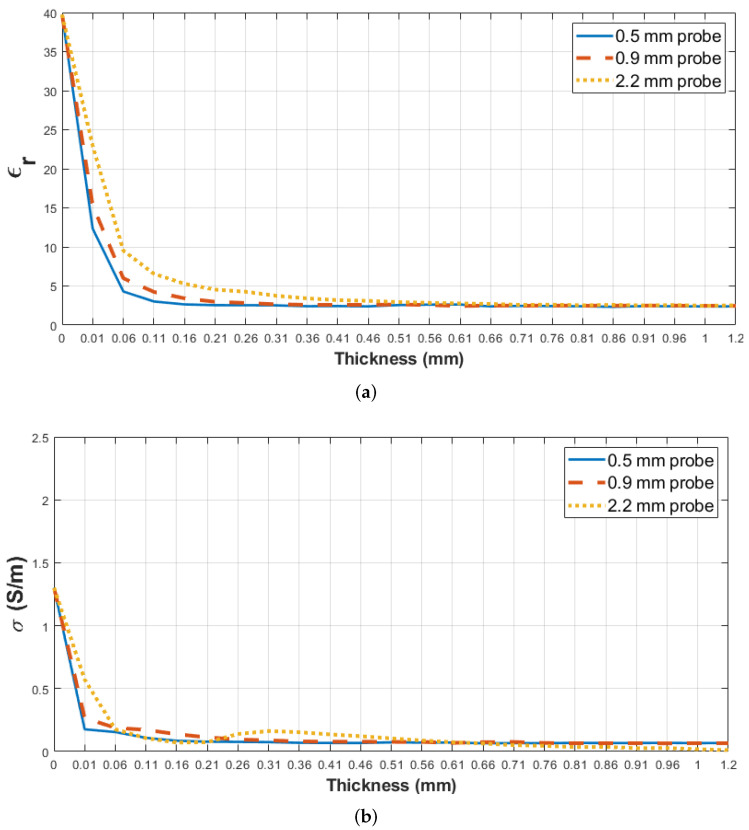
Retrieved dielectric property changes obtained from the simulated skin phantoms–olive oil liquids with 0.5, 0.9 and 2.2 mm probes: (**a**) retrieved relative permittivity change and (**b**) retrieved conductivity change as a function of second layer thickness (d) below the probe tips at 4 GHz.

The proposed double-layered skin–olive oil sample was designed and simulated with the 0.5, 0.9 and 2.2 mm diameter probes. The retrieved relative permittivity and conductivity from the simulation results as a function of the second layer thickness (d) are shown in Figure 4. When the thickness (d) on the graph is equal to 0, it refers to the probe tip being terminated by the first layer. Even though the sensing depth was examined from 0 to 5 mm thicknesses of the second layer, the (thickness) axes in Figure 4 were plotted from 0 to 1.2 mm to clearly depict the graph. This is due to the rapid changes in dielectric properties at smaller thicknesses. In order to plot and demonstrate the rapid dielectric property changes for sensing depth analysis, critical thickness values (0, 0.01–1 mm with 0.05 mm increments and 1.2 mm) were selected for the thickness axes. Therefore, the thickness axes in Figure 4 are not linearly or logarithmically plotted. For the 0.5 mm-diameter probe, a retrieved dielectric property approximately close to that of olive oil was reached when the thickness of the second layer was 0.31 mm. However, at the same thickness (d = 0.31 mm), the dielectric property value acquired via the 2.2 mm diameter probe was 80% higher than the value of olive oil. Therefore, it can be inferred that at the same thickness, the probe with a bigger aperture size can sense the first layer (skin tissue); thus, a probe with a larger aperture size is expected to have a larger SD.

In [8], two different probes with 1 and 3 mm diameter aperture sizes were used to define the proper thickness of the sample that does not affect the measurement of relative permittivity. A double-layered sample configuration including porcine muscle as the bottom layer and porcine fat as the upper layer was utilized. The proper thicknesses for the porcine fat layer were determined to be 1 and 3 mm for the 1 and 3 mm diameter probe, respectively. The study defined the term “proper thickness” for a double-layered biological tissue, and their results indicated that the probe’s aperture size is proportional to the SD.

In [10], the sensing depth from the simulation and experimental results of the 2.2 and 3.58 mm diameter probes for glass–ethanol, glass–methanol and glass–deionized water (d.water) double-layered configurations was presented. Higher SD values for the probe with the larger aperture size from both the simulation and experiment results irrespective of the sample configuration were obtained. Furthermore, ±10% was chosen as the acceptable level of error in the real and imaginary parts of the complex permittivity.

In [11], from the simulation results, the effective penetration depths (20% reduction from expected dielectric property value) were found to be 0.158, 0.483 and 0.866 mm for the 1.19, 3.58 and 6.35 mm diameter probes, respectively. The obtained 20% change in relative permittivity from our simulation results for the 0.5, 0.9 and 2.2 mm probes was 0.11, 0.21, 0.46 mm, respectively. The effective penetration depth obtained in [11] is higher than the sensing depth in our work for the same percentage change in relative permittivity. This difference is related to the different probe dimensions (inner and outer conductor diameters) used in the simulations. Even though different values were obtained from both works, the results still indicate that the sensing depth/effective penetration depth increases as the probe aperture size increases.

In this work, the thicknesses of three different levels of percentage increase for the retrieved relative permittivity of skin–olive oil double-layered configurations at 4 GHz with three different probes are presented and listed in Table 9. According to the results, the increase in retrieved dielectric properties from 5% to 80% was slower for the 2.2 mm-diameter probe. Based on these results, the 2.2 mm diameter probe is more sensitive to the dielectric property of the skin phantom layer below the olive oil layer. These findings suggest that sensing depth is dependent on the probe aperture; that is, the SD increases with the increase in probe aperture for such a configuration.

**Figure 5:**
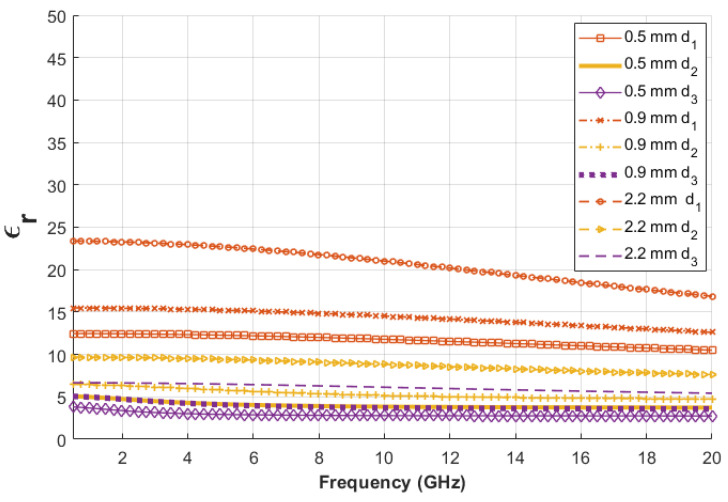
The comparison of skin–olive oil retrieved relative permittivity through simulation of 0.5, 0.9 and 2.2 mm diameter probes as a function of frequency when the second layer thicknesses (d${}_{1}$, d${}_{2}$ and d${}_{3}$) are 0.01, 0.06 and 0.11 mm.

Lastly, to analyze the critical variation of the dielectric property, the thickness of the olive oil layer was set to d${}_{1}=0.01$, d${}_{2}=0.06$ and d${}_{3}=0.11$ and simulated with the three different probes. The results of the dielectric properties obtained from the simulations at the given olive oil thicknesses are plotted with respect to frequency in Figure 5. Additionally, the dielectric property results based on the olive oil thickness and probe apertures are listed in Table 10. As seen from the table, the dielectric properties of first layer (skin) have a greater effect on the double-layered results performed with the 2.2 mm diameter probe.

As shown in Table 10, at 0.11 mm thickness of olive oil, the retrieved relative permittivities are 3.02, 4.24 and 6.56 for 0.5, 0.9 and 2.2 mm diameter probes, respectively. Simulated results acquired by the 0.5 mm diameter probe converged to the dielectric properties of olive oil at the smallest thickness under the same conditions.

## 5. Conclusions

The OECP method is superior to other dielectric property measurement techniques for the dielectric property characterization of biological tissues due to its measurement simplicity. However, the method suffers from equipment and tissue-related errors. Minimizing the effect of skin tissue heterogeneity and the sensing depth of the probe is an important amendment for classifying skin tissue anomalies and the detection of skin cancer through dielectric property discrepancy. Therefore, there is a need to understand the effect of sensing depth in order to implement the appropriate probe for the application. Towards this end, the sensing depths of three different probe aperture sizes were compared in this work. Probes with 0.5, 0.9 and 2.2 mm diameters were simulated with a double-layered skin tissue and olive oil sample configuration. The experimental validation was performed with the 2.2 mm diameter probe with the skin phantom and olive oil configuration. The obtained results show that the sensing depth is proportional to the probe aperture, and a smaller probe aperture can potentially aid in diminishing the heterogeneity of the tissue and allow the accurate dielectric property characterization of thin skin tissue layers. As an example, a 5% increase in retrieved relative permittivities was obtained at 0.31, 0.56 and 0.71 mm thicknesses for 0.5, 0.9 and 2.2 mm diameter probes, respectively. Based on these results, we can state that using a 0.5 mm diameter probe can provide more accurate results when a thin skin layer measurement is performed. However, the membrane or any tissue fluid that may be present on the tissue surface during measurement with a 0.5 mm diameter probe may affect the measurement result more than expected. Therefore, measurement protocols should be established to prepare the measurement surface for tissues that consist of a multi-layered structure or that may have a membrane or any other layer on the measurement surface. Alternatively, tissue heterogeneity can be determined using probes with multiple aperture sizes.

## Figures and Tables

**Table 1:** Simulated probe dimensions with different aperture sizes.

Outer Radius (mm)	Inner Radius (mm)	Dielectric Type	$\epsilon_{r}$
0.25	0.075	PTFE	2.1
0.45	0.134		
1.1	0.328		

**Table 2:** The relative permittivity and conductivity of skin and olive oil used to create the double-layered configurations at 4 GHz for simulations.

Layers	Materials	Relative Permittivity ($\epsilon_{r}$)	Conductivity (S/m)
First layer [15]	Skin	32.89	2.29
Second Layer [16]	Olive oil	3.00	0.04

**Table 3:** The relative permittivity and conductivity of skin and olive oil in double-layered configuration for sensing depth experiment at 4 GHz.

Materials	Relative Permittivity ($\epsilon_{r}$)	Conductivity (S/m)
Skin	34.17	4.00
Olive oil	2.67	0.02

**Table 4:** Retrieved relative permittivity change of skin–olive oil double-layered configurations with 2.2 mm diameter probe at 0.5, 2, 4, 10 and 20 GHz frequency points.

Frequency	Thickness for 5% Increase	Thickness for 20% Increase	Thickness for 80% Increase
0.5	1	0.86	0.36
2	0.76	0.56	0.26
4	0.71	0.46	0.21
10	0.66	0.41	0.21
20	0.66	0.36	0.16

**Table 5:** Measured relative permittivity change of skin–olive oil double-layered configurations with 2.2 mm diameter probe at 0.5, 2, 4, 6 and 8 GHz frequency points.

Frequency	Thickness for 5% Increase	Thickness for 20% Increase	Thickness for 80% Increase
0.5	0.81	0.67	0.22
2	0.81	0.67	0.22
4	0.81	0.67	0.22
6	0.81	0.67	0.20
8	0.81	0.67	0.22

**Table 6:** Retrieved relative permittivity change of skin–olive oil double-layered configurations with 0.9 mm diameter probe at 0.5, 2, 4, 10 and 20 GHz frequency points.

Frequency	Thickness for 5% Increase	Thickness for 20% Increase	Thickness for 80% Increase
0.5	0.66	0.21	0.11
2	0.56	0.26	0.11
4	0.56	0.21	0.11
10	0.56	0.21	0.11
20	0.51	0.21	0.06

**Table 7:** Retrieved relative permittivity change of skin–olive oil double-layered configurations with 0.5 mm diameter probe at 0.5, 2, 4, 10 and 20 GHz frequency points.

Frequency	Thickness for 5% Increase	Thickness for 20% Increase	Thickness for 80% Increase
0.5	0.31	0.11	0.06
2	0.31	0.16	0.06
4	0.31	0.11	0.06
10	0.31	0.11	0.06
20	0.31	0.11	0.06

**Table 8:** List of literature studies on different aperture sizes, sample configurations and different terminologies to define the sensing depth of the OECP.

Study	Definition	Type	Aper. Size (mm)	Sam. Config.	Sample’s $\epsilon^{\prime }$	Depth (mm)
[8]	“Proper thickness of target-sample” is the thickness of the target sample where the influence of the non-targeted region is no longer sensed by the probe.	Exp.	1.0	Porcine muscle–porcine fat	Porcine muscle $\epsilon^{\prime }$ = ∼52 porcine fat $\epsilon^{\prime }$ = ∼8 at 0.5 GHz.	1.0
			3.0			3.0
[10]	“Sensing volume” was defined by monitoring the changes in the $S_{11}$ between ±0.5–±1.0 error which is ±10% error in terms of the $\epsilon^{\prime }$ and $\epsilon^{\prime \prime }$.	Sim.	2.2	Glass–ethanol	Glass $\epsilon^{\prime }$ = 4.82, ethanol $\epsilon^{\prime }$ = 9.6, methanol $\epsilon^{\prime }$ = 24.2, d.water $\epsilon^{\prime }$ = 79.2 at 3 GHz. Below the glass beaker, there is an epoxy stand $\epsilon^{\prime }$ = 4.0 at 5 GHz.	0.5–1.0
				Glass–methanol		1.0–1.5
				Glass–d.water		1.25–1.5
			3.58	Glass–ethanol		1.0–1.5
				Glass–methanol		∼1.75
				Glass–d.water		∼2.5
		Exp.	2.2	Glass–ethanol		0.75–1.0
				Glass–methanol		1.0–1.5
				Glass–d.water		∼1.5
			3.58	Glass–ethanol		1.25–1.5
				Glass–methanol		∼2.25
				Glass–d.water		∼2.5–3.0
[11]	“Effective penetration depth” was defined a when 20% error is observed between the measured and expected linearly changing $\epsilon^{\prime }$.	Sim	1.19	Acrylic–0.9% saline	Acrylic $\epsilon^{\prime }$ = 3.0 0.9% saline $\epsilon^{\prime }$ = 78.1 d.water $\epsilon^{\prime }$ = 78.8 at 2 GHz.	0.158
			3.58			0.483
			6.35			0.866
		Exp.	18.0	Acrylic–d.water		1.84
			21.0			2.74
			2.16	Teflon–d.water	Teflon $\epsilon^{\prime }$ = 2.1 d.water $\epsilon^{\prime }$ = 78.8 at 2 GHz.	0.28
This work	“Sensing depth” is defined as when 5% change in the targeted $\epsilon^{\prime }$ is observed.	Sim.	2.2	Skin tissue–olive oil	Skin tissue $\epsilon^{\prime }$ = 32.89, olive oil $\epsilon^{\prime }$ = 3.0 at 4 GHz	0.71
			0.9			0.56
			0.5			0.31
		Exp.	2.2	Skin-mimicking phantom–olive oil	Skin-mimicking phantom $\epsilon^{\prime }$ = 34.7, olive oil $\epsilon^{\prime }$ = 2.67 at 4 GHz	0.81

**Table 9:** Thickness of three different increase levels for retrieved relative permittivity of skin–olive oil double-layered configurations at 4 GHz with three different probes.

Probe Aperture (mm)	Thickness for 5% Increase	Thickness for 20% Increase	Thickness for 80% Increase
0.5	0.31	0.11	0.06
0.9	0.56	0.21	0.11
2.2	0.71	0.46	0.21

**Table 10:** Three different thicknesses and retrieved relative permittivity of skin–olive oil double-layered configurations at 4 GHz with three probes.

Thickness (mm)	Relative Permittivity ($\epsilon_{r}$)		
	0.5 mm Probe	0.9 mm Probe	2.2 mm Probe
0.01	12.34	15.32	22.95
0.06	4.29	5.98	9.52
0.11	3.02	4.24	6.56

## Data Availability

Not applicable.

## References

[1] Gabriel C., Gabriel S., Corthout Y.E. (1996). The dielectric properties of biological tissues: I. Literature survey. Phys. Med. Biol..

[2] Jafary-Asl A., Smith C. Biological dielectrics in electric and magnetic fields. Proceedings of the Conference on Electrical Insulation & Dielectric Phenomena-Annual Report 1983.

[3] Yilmaz T., Ates Alkan F. (2020). In vivo dielectric properties of healthy and benign rat mammary tissues from 500 MHz to 18 GHz. Sensors.

[4] Lazebnik M., Popovic D., McCartney L., Watkins C.B., Lindstrom M.J., Harter J., Sewall S., Ogilvie T., Magliocco A., Breslin T.M. (2007). A large-scale study of the ultrawideband microwave dielectric properties of normal, benign and malignant breast tissues obtained from cancer surgeries. Phys. Med. Biol..

[5] Yilmaz T., Kılıç M.A., Erdoğan M., Çayören M., Tunaoğlu D., Kurtoğlu İ., Yaslan Y., Çayören H., Arıkan A.E., Teksöz S. (2016). Machine learning aided diagnosis of hepatic malignancies through in vivo dielectric measurements with microwaves. Phys. Med. Biol..

[6] Mirbeik-Sabzevari A., Ashinoff R., Tavassolian N. (2017). Ultra-wideband millimeter-wave dielectric characteristics of freshly excised normal and malignant human skin tissues. IEEE Trans. Biomed. Eng..

[7] Mohammed B., Naqvi S., Manoufali M., Bialkowski K., Abbosh A. Changes in epidermal dielectric properties due to skin cancer across the band 1 to 50 GHz. Proceedings of the 2018 Australian Microwave Symposium (AMS).

[8] Hwang H., Yim J., Cho J.W., Cheon C., Kwon Y. 110 GHz broadband measurement of permittivity on human epidermis using 1 mm coaxial probe. Proceedings of the IEEE MTT-S International Microwave Symposium Digest.

[9] La Gioia A., Porter E., Merunka I., Shahzad A., Salahuddin S., Jones M., O’Halloran M. (2018). Open-ended coaxial probe technique for dielectric measurement of biological tissues: Challenges and common practices. Diagnostics.

[10] Hagl D.M., Popovic D., Hagness S.C., Booske J.H., Okoniewski M. (2003). Sensing volume of open-ended coaxial probes for dielectric characterization of breast tissue at microwave frequencies. IEEE Trans. Microw. Theory Tech..

[11] Meaney P.M., Gregory A.P., Seppälä J., Lahtinen T. (2016). Open-ended coaxial dielectric probe effective penetration depth determination. IEEE Trans. Microw. Theory Tech..

[12] La Gioia A., Santorelli A., O’Halloran M., Porter E. (2020). Predicting the Sensing Radius of a Coaxial Probe Based on the Probe Dimensions. IEEE Trans. Antennas Propag..

[13] Aydinalp C., Joof S., Yilmaz T. (2021). Towards Non-Invasive Diagnosis of Skin Cancer: Sensing Depth Investigation of Open-Ended Coaxial Probes. Sensors.

[14] Aydinalp C., Joof S., Yilmaz T. (2021). Towards Accurate Microwave Characterization of Tissues: Sensing Depth Analysis of Open-Ended Coaxial Probes with Ex Vivo Rat Breast and Skin Tissues. Diagnostics.

[15] Alekseev S., Ziskin M. (2007). Human skin permittivity determined by millimeter wave reflection measurements. Bioelectromagnetics.

[16] Šegatin N., Pajk Žontar T., Poklar Ulrih N. (2020). Dielectric Properties and Dipole Moment of Edible Oils Subjected to ‘Frying’Thermal Treatment. Foods.

[17] Aydinalp C., Joof S., Yilmaz T. Sensing depth analysis of open-ended coaxial probe for skin cancer detection. Proceedings of the 2019 23rd International Conference on Applied Electromagnetics and Communications (ICECOM).

[18] Porter E., O’Halloran M. (2017). Investigation of histology region in dielectric measurements of heterogeneous tissues. IEEE Trans. Antennas Propag..

